# Flavor omics approach of dried *Lyophyllum decastes* mushroom using GC-IMS, GC-O-MS, *E*-nose, *E*-tongue, and multiple factor analysis

**DOI:** 10.1016/j.fochx.2026.103925

**Published:** 2026-04-26

**Authors:** Min Sun, Jingbo Shen, Yan Liu, Tao Feng, Shiqing Song, Chuang Yu, Huatian Wang, Lingyun Yao, Min Deng

**Affiliations:** a Faculty of Flavour Fragrance and Cosmetics, Shanghai Institute of Technology, Shanghai 201418, China; b School of Hotel Management and Culinary Arts, Shanghai Institute of Tourism, Shanghai 201418, China

**Keywords:** Dried *Lyophyllum decastes*, Aroma profile, Volatile organic compound, Non-volatile organic compound, Taste, Multiple factor analysis

## Abstract

Dried *Lyophyllum decastes*, which possesses a diversity of varieties and a distinctive flavor profile, is highly appreciated by consumers. Electronic nose, electronic tongue, GC-IMS, and GC-O-MS were used to investigate the flavor of four dried L. *decastes* samples. The aroma of dried L. *decastes* could be classified into seven categories, among which rancid, sweet, and floral constituting the dominant notes. Taste attributes varied significantly among samples, especially, the SH dried L. *decastes* exhibited prominent umami and sweet flavors due to its high content of sweet amino acids (4577.62 mg/100 g) and reducing sugars (20.13 mg/g). Multiple factor analysis indicated that electronic nose coupled with electronic tongue could effectively distinguish different samples (RV = 0.945). Furthermore, non-volatile compounds were found to correlate with aroma properties. This study provided valuable analytical data for characterizing and distinguishing the flavor profiles of different dried L. *decastes*, demonstrating the effectiveness of multi-technique applications in flavor characterization.

## Highlights

• The aroma profile of dried L. *decastes* was first classified into seven categories.
• The rancid odor of dried L. *decastes* may correlate with acidic compounds content.
• MFA explores connections in multi-instrument/multi-variable data of dried L. *decastes*.
• Combined E-nose and E-tongue effectively distinguish different dried L. *decastes*.

## 1 Introduction

*Lyophyllum decastes*, also known as the lotus leaf umbrella mushroom or lotus leaf fungus, is widely favored by consumers owing to its delicious flavor, distinctive taste, and rich nutritional value **(**Yang et al., 2022**)**. Besides that, *L. decastes* possess extensive bioactivities such as anti-oxidation, anti-diabetes, anti-proliferative, and anti-obesity properties **(**Li et al., 2024**)**. As a renowned comestible and therapeutic fungus, *L. decastes* has exhibited strong expansion trend in Chinese edible mushroom market with an average growth rate of approximately 75.94% per year over the past decade **(**Li et al., 2024**;** Ling et al., 2025**)**. For edible mushroom, the flavor property could significantly influence consumer's preference and acceptance, which is determined by the combination of volatile and non-volatile compounds **(****Sun et al., 2020****)**. In recent years, studies have reported that taste profiles of fresh L. *decastes* were correlated with their production regions, and volatile flavor compounds of L. *decastes* could be greatly altered by drying process **(**Yang et al., 2022**;** Zhang et al., 2026**)**. However, there is currently a lack of systematic research on the aroma and taste of dried L. *decastes* from different main production regions in China.

The concept of flavor omics was first proposed in 2008 and represents a significant advancement in characterization of the components that affect the flavor of food. These components are combined with chemometrics and other techniques to extract, screen, and identify key compounds that affect flavor **(**Cai et al., 2024**;** Pérez-Jiménez et al., 2021**)**. The common analytical techniques employed in flavor omics include intelligent sensory analysis technology such as electronic nose (*E*-nose) and electronic tongue (E-tongue), and chromatography–mass spectrometry technology such as gas chromatography–mass spectrometry (GC–MS) and high performance liquid chromatography-mass spectrometry (HPLC-MS) **(**Hou et al., 2021**;** Wei et al., 2023**)**. In terms of aroma compound analysis, GC–MS and gas chromatography-ion mobility spectrometry (GC-IMS) are particularly valued for their low sample consumption, high sensitivity, and rapid analysis, enabling precise profiling of volatile flavor compounds **(**Yun et al., 2021**)**. Further, intelligent sensory analysis technology employs multi-sensor to simulate human olfactory and gustatory responses has been widely used in flavor omics researches **(**Tibaduiza et al., 2024**)**. The sensors offer the benefits of objectivity, accuracy, simplicity, and economy method on elucidating various food flavor characteristics **(**Yang et al., 2016**)**. In recent years, flavor omics have been widely applied to analyze flavor differences in various food products like yogurt, baijiu, coffee and edible mushrooms **(**Gao et al., 2023; Han et al., 2024; Hou et al., 2024; **Li, Han, et al., 2025**; Zhu et al., 2024**)**. As example, flavor profiles of several edible mushrooms such as *Matsutake*, *Pleurotus ostreatus*, and *Lentinus edodes* have been well elucidated by aforementioned analytical techniques (Yan et al., 2025**;** Zhang et al., 2021**;** Zhong et al., 2024**)**.

For flavor omics approaches, data analysis is of great importance on facing numerous challenges when dealing with complex flavor datasets of various foods. Hence, many chemometric techniques (i.e., multivariate statistical methods) have been developed to explore relationships among analytical, sensory, and spectroscopic data, such as principal component analysis (PCA), multiple factor analysis (MFA), and partial least squares regression (PLSR) **(**Li et al., 2021**;** Zielinski et al., 2014**)**. Among which, MFA is a multivariate statistical method designed for the analysis of multiple data sets, particularly suitable for the joint analysis of multi-instrument and multi-type variables, including both qualitative and quantitative data **(**Sick et al., 2023**;** Yu et al., 2024**)**. For example, the MFA method has been successfully applied to integrate sensory data, physicochemical properties, and target volatile compounds in Chinese fermented tofu in dimensionality reduction and visualization for large-scale data analysis **(**He & Chung, 2019**)**.

Several studies have focused on physicochemical and flavor changes of L. *decastes* upon different drying methods **(**Yang et al., 2022**)**. Nevertheless, the flavor profile of dried L. *decastes* is primarily determined by multiple factors including variety, cultivation conditions, and drying techniques. In order to further characterizes the flavor profile of dried *L. decastes*, four representative mushroom types were sampled due to their strong online sales performance and distinct geographical origins, with their flavor compounds and characteristics of each sample were evaluated by combining GC–MS, GC-IMS, *E*-nose, and E-tongue technologies. Furthermore, MFA was performed on the comprehensive instrumental data to achieve systematic identification and flavor characterization of different dried L. *decastes* products.

## 2 Materials and methods

### 2.1 Samples and chemicals

Four commercial dried L. *decastes* products were used in this study, including Sample SH collected from Shanghai Rongmei Agricultural Technology Co., Ltd. (Shanghai, China), Sample GZ collected from Guizhou Guangqian Tongxin Biomedical Technology Co., Ltd. (Guizhou, China), Sample YN collected from Kunming Youxuan Yungong Food Co., Ltd. (Yunnan, China), and Sample FJ collected from Fujian Xueertang Biotechnology Co., Ltd. (Fujian, China). Except for production regions, all samples were of the same cultivar and processed via hot-air drying. All samples were vacuum-sealed and stored at room temperature in a dry and well-ventilated environment. Prior to analysis, the dried L. *decastes* were ground using a pulverizer and passed through a sieve to obtain coarse powder with a particle size ranging approximately from 80 to 150 mesh.

Standard *n*-alkane (C_7_–C_30_) compounds were purchased from Sigma-Aldrich (Shanghai, China). Standard *n*-ketone (C_4_–C_9_) were purchased from Sinopharm Chemical Reagent Co., Ltd. (Beijing, China). 5′-Nucleotides and organic acid standards were obtained from Sigma Chemical Company (St. Louis, MO, USA), and amino acid standards were purchased from Wako Pure Chemical Industries, Ltd. (Chuo-ku, Japan). Standard substances of nonanal, isobutyric acid, limonene, benzyl alcohol, benzaldehyde, 1-octen-3-ol and 2-pentylfuran were purchased from Titan Technology Co., Ltd. (Shanghai, China) and Macklin Biochemical Co., Ltd. (Shanghai, China). All other reagents and solvents were analytically pure and purchased from Sinopharm Chemical Reagent Co., Ltd. (Shanghai, China).

### 2.2 Physicochemical properties of different dried L. decastes samples

The moisture content of dried L. *decastes* was analyzed using an XY-110 MW-T rapid moisture analyzer (Changzhou Lucky Electronic Equipment Co., Ltd., Changzhou, China). The total protein content was determined using the Kjeldahl method, and total ash content was measured by weighing the residue after incineration at 550 °C for 24 h **(**Jacinto-Azevedo et al., 2021**)**. Reducing sugar content was determined by the DNS method with minor modifications, while total sugar content was measured by DNS method after acid hydrolysis **(**Turfan et al., 2018**)**. For polysaccharides analysis, the polysaccharides were isolated and purified from the water extract of dried L. *decastes* by ethanol precipitation, deproteinization, and dialysis **(**Xu et al., 2015**)**.

### 2.3 Aroma characteristics of dried L. decastes detected by *E*-nose

E-nose analysis was performed using the PEN3 electronic nose system (AIRSENSE, Germany). The electronic nose probe consists of 10 metal oxide sensors with distinct performance characteristics (W1C, W5S, W3C, W6S, W5C, W1S, W1W, W2S, W2W, W3S), exhibiting sensitivity to various compounds. The analysis was conducted with slight modifications based on the method described previously **(**Qin et al., 2020**)**. Briefly, 2 g of sample was placed in a 25 mL sample vial, and the electronic nose probe, composed of 10 metal oxide sensors with different sensing properties, was inserted into the headspace of the vial for measurement. The detection parameters were as follows: measurement time, 80 s; pre-sampling time, 5 s; sampling interval, 1 s per group; flush time, 300 s; zeroing time, 5 s; carrier gas flow rate, 400 mL/min. Before testing the next sample, the system was purged with treated, dry and clean air. All measurements were repeated four times and the last three data were collected for analysis.

### 2.4 Headspace-gas chromatography-ion mobility spectrometry (HS-GC-IMS)

The GC-IMS system was composed of an Agilent 490 gas chromatograph (Agilent Technologies, USA), an automatic sampler (CTC Analytics AG, Zwingen, Switzerland) and an IMS instrument (FlavourSpec®, Gesellschaft für Analytische Sensorsysteme mbH, Dortmund, Germany). Nitrogen gas (≥99.99%) was used as the carrier gas, and chromatographic separation was performed using an MXT-WAX column (30 m × 0.53 mm, 1.0 μm). Identification of volatile compounds was conducted with slight modifications based on the previously reported method **(**He et al., 2024**)**. Approximately 2 g of each sample was weighed into a 20 mL headspace vial and incubated at 45 °C for 15 min. The syringe was heated to 85 °C to extract headspace gas (500 μL), which was automatically injected by the sampler in splitless mode. The gas flow rates were set as follows: 2 mL/min for 0–2 min, 10 mL/min for 2–10 min, and 100 mL/min for 10–20 min, maintained at 100 mL/min for 40 min (20–59 min). After chromatographic separation, volatile compounds were ionized in the ionization chamber. The resulting ions were driven through a 9.8 cm drift tube maintained at 45 °C. Nitrogen gas (99.99%) was used as the drift gas with a flow rate of 150 mL/min.

The retention index (RI) and drift time (DT) of each compound were calculated using the standard *n*-ketone (C_4_–C_9_) as an external reference **(**Yao et al., 2021**)**. Identification was further confirmed by searching the Laboratory Analytical Viewer (LAV, G.A.S., Dortmund, Germany) and the GC-IMS database (Gesellschaft für Analytische Sensorsysteme mbH, Dortmund, Germany).

### 2.5 GC–MS and gas chromatography-olfactometry (GC-O) analysis

#### 2.5.1 GC–MS analysis

The headspace solid-phase microextraction (HS-SPME) coupled with GC–MS was employed for further analysis of the volatile compounds of dried L. *decastes* based on the reported methods with slight modification **(**Chen et al., 2021**)**. Samples were analyzed by an Agilent 8860 A gas chromatograph and a 5977B mass spectrometer (Agilent Technologies, Santa Clara, CA, USA). Firstly, 1 g of dried *L. decastes* powder was poured into a headspace bottle, and mixed with 30 μL of o-dichlorobenzene (100 ppm) as an internal standard. The sample was equilibrated in a 60 °C water bath for 20 min, followed by extraction with an SPME fiber for 30 min. The fiber was then immediately inserted into the GC–MS injection port for desorption at 250 °C for 6 min. The sample heating program was as follows: the initial temperature was set at 40 °C and maintained for 3 min; the temperature was then increased to 120 °C at a rate of 5 °C/min and held for 2 min; finally, it was raised to 230 °C at a rate of 3 °C/min and maintained for 5 min.

The volatile components of dried L. *decastes* were separated by an HP-INNOWAX column (60 m × 0.25 mm × 0.25 μm, Agilent Technologies, USA), with helium (>99.99%) used as the carrier gas at a flow rate of 1.68 mL/min and a split ratio of 5:1. The mass spectrometer was operated in electron ionization mode with an ionization energy of 70 eV and an ion source temperature of 230 °C. The mass scanning was conducted in the range of 30–450 *m*/*z* in the full-scan mode. Each compound was identified by mass spectra and compared with the NIST Mass Library (version 2020), with retention indices (RIs) were calculated for further identification through comparing with reference values (*https://webbook.nist.gov/chemistry/*) **(**Yao et al., 2023**)**.

The concentrations of volatile compounds were determined by comparing with the concentration of the internal standard (1,2-dichlorobenzene). The calculation formula was as follows:

$$C=C_{s}\times V_{s}\times A_{i}/\left(m_{0}\times A_{s}\right)$$

where *C* was the concentration of volatile substances in the sample, mg/kg; *C*_s_ was the internal standard concentration, mg/kg; V_s_ was the volume of internal standard, μL; *m*_0_ was the mass of the sample, g; *A*_i_ and *A*_s_ were the peak areas of the internal standard and volatile substances respectively **(**Yao et al., 2024**)**.

#### 2.5.2 GC-O analysis

Aroma evaluation of the generated volatile compounds was performed using GC-O equipped with an ODP-2 olfactory detection port (Gerstel, Mülheim an der Ruhr, Germany). The chromatographic conditions were identical to those described for GC–MS, with nitrogen gas (>99.99%) as the carrier gas. During the GC-O process, the effluent was split between the detector and the sniffing port at a ratio of 1:1 to determine the retention times, aroma characteristics, and intensities of the volatile compounds respectively. Prior to GC-O analysis, sensory assessors were selected, trained, and assessed (e.g.,discrimination test and intensity ratings) according to **ISO 8586**. Panelists consisted of five females and five males (aged from 20 and 30) from Shanghai Institute of Technology, Shanghai, China, who have been trained in similar sensory experiments for at least 1 year. Seven aroma descriptors were identified: fat, rancid, citrus, flower, sweet, mushroom, and roasted nut. The corresponding reference standards for these attributes were nonanal, isobutyric acid, limonene, benzyl alcohol, benzaldehyde, 1-octen-3-ol, and 2-pentylfuran, respectively. Panelists was required to describe the smell of the mushroom samples after familiarizing themselves with the scents of the seven fragrances. The aroma intensity of each note was evaluated using 0 points (none) to 9 points (strong) method as described in previous works **(**Feng et al., 2021**;** Yao et al., 2021**)**. Sensory evaluation was conducted with the approval of the Ethics Committee of the Shanghai Institute of Technology (Shanghai, China), and all panelists signed informed consent forms. All dried L. *decastes* samples were evaluated in triplicate.

### 2.6 Taste characteristics of dried L. decastes detected by *E*-tongue

The evaluation of sourness, sweetness, bitterness, saltiness, umami, astringency, aftertaste-B (bitter aftertaste), aftertaste-A (astringent aftertaste), and richness in dried L. *decastes* were analyzed using the INSENT SA-402B electronic tongue system (INSENT, Atsugi City, Japan). Sample preparation involved grinding the dried L. *decastes*, adding deionized water at a 1:40 ratio, soaking for half an hour, followed by boiling for another 30 min and centrifugation to collect the supernatant. The sample solution was then taken into electronic tongue specialized beakers and tested four times using the SA402B electronic tongue. The procedure was set to 30 s for taste collection, aftertaste collection time of 30 s, and cleaning time of 300 s. All measurements were repeated four times and the last three data were collected for analysis.

### 2.7 Non-volatile flavor compound analysis

#### 2.7.1 Determination of free amino acids

Amino acid content was determined with slight modifications based on the method described previously **(**Liu et al., 2014**)**. A 2 mL sample was mixed with 2 mL of 10 g/L sulfosalicylic acid and 1 mL of 10 g/L EDTA, followed by ultrasonic extraction for 60 min. After extraction, 2 mL of the mixed solution was dried under a nitrogen stream and then redissolved in distilled water to a final volume of 25 mL. The redissolved solution was filtered through a 0.22 μm membrane filter and subsequently analyzed.

The parameters of the fully automated amino acid analyzer were as follows: sample volume, 20 μL; pump flow rate range, 0–0.9 mL/min; column temperature, 57 °C; reactor temperature, 135 °C; maximum pressure, 30 MPa; injection volume, 8 μL; and detection wavelength, 570 nm. After analyzing the samples and comparing them with amino acid standards, the instrument automatically generated the analytical reports.

#### 2.7.2 HPLC analysis

Following reported method with slight modification **(**Du et al., 2024**)**, analysis was performed using an HPLC system (Agilent Technologies, Palo Alto, CA, USA) equipped with a 10 μL sample injection loop. For organic acids detection, 1 g of dried L. *decastes* powder was weighed and placed into a 50 mL centrifuge tube, to which 25 mL of ultrapure water was added. The mixture was then homogenized and sonicated for 30 min. Afterward, the solution was filtered and transferred to a 25 mL volumetric flask, where the volume was adjusted to the calibration mark with water. The solution was then mixed thoroughly and filtered through a 0.22 μm membrane filter. Chromatographic conditions: An LP-C18 column (4.6 mm × 250 mm × 5 μm) was used. Mobile phase A was a 0.1% phosphoric acid aqueous solution, and mobile phase B was acetonitrile. The column temperature was maintained at 35 ± 2 °C, with a detection wavelength of 210 nm and a flow rate of 0.5 mL/min. The injection volume was 10 μL.

For 5′-nucleotides detection, 300 mg of dried L. *decastes* powder was weighed and added to 10 mL of ultrapure water. The mixture was extracted in a boiling water bath for 10 min, cooled to room temperature, and then centrifuged at 12,000 r/min for 15 min. The supernatant was collected and passed through a 0.22 μm filter membrane. Chromatographic conditions: An Ultimate AQ-C_18_ column (250 mm × 4.6 mm × 5 μm) was used, with a mobile phase of 10 mmol/L KH_2_PO_4_ buffer at pH 4.68. The column temperature was set at 30 °C, and the detection wavelength was 249 nm. The flow rate was 1.0 mL/min, and the injection volume was 10 μL.

### 2.8 Statistical analysis

The HS-GC-IMS instrument analysis software is mainly composed of Laboratory Analytical Viewer (LAV, G.A.S., Dortmund, Germany), three plug-ins (G.A.S.) and GC × IMS Library Search. Results were expressed as mean ± standard deviation. All the measurements were performed in triplicate. Mean values were considered significantly different at *P* < 0.05. Differences between means were determined using ANOVA and least significant difference (LSD) test using SPSS 26.0 software (SPSS Inc., USA). Radar charts were drawn using Origin 2022 software (OriginLab Co., Northampton, MA, USA). PCA was performed by SIMCA 14.1 version (Umetrics, Umea, Sweden). MFA was performed by XLSTAT 2019 version (Addinsoft, New York, USA).

## 3 Results and discussion

### 3.1 Physicochemical properties of different dried L. decastes samples

The physicochemical property of four dried L. *decastes* was revealed in Table 1. The moisture content ranged from 77.13 to 145.34 mg/g, and the ash contents of the GZ and YN samples were 112.64 mg/g and 110.27 mg/g respectively **(**Table 1**)**. The ash content of dried L. *decastes* here (ranged from 9.83% to 11.26%) was higher than that value (6.39% ∼ 8.45%) of other production regions in China including Jiangsu and Hebei **(****Li, Yang, et al., 2025****;** Zhang et al., 2026**)**. In terms of total protein content, the value ranged from 19.69% to 24.31% with SH sample revealed to be the highest (243.12 mg/g), which was similar to the reported value (20.25% ∼ 27.88%) of other production regions **(****Li, Han, et al., 2025****;** Zhang et al., 2026**)**. Total sugar is another main component of dried L. *decastes*, with its content ranged from 248.35 to 387.23 mg/g and the polysaccharide content ranged from 74.45 to 112.09 mg/g observed (Table 1), and these values were coincided with reported values of other production regions (2.86% ∼ 10.30%) **(****Li, Han, et al., 2025****;** **Wu, Liang, et al., 2025****)**. Nevertheless, the reducing sugar content of the four samples was relatively low, ranging from 12.27 to 20.45 mg/g **(**Table 1**)**. These results indicated that the physicochemical and nutritional components of commercially available dried L. *decastes* samples varied significantly, which could be mainly attribute to differences in production regions and cultivars.

**Table 1:** Comparison of physicochemical properties (moisture, protein, polysaccharide, ash content, total sugar, reducing sugar) of dried L. *decastes*.

Sample Content(mg/g)	SH	GZ	YN	FJ
Moisture	77.13 ± 1.84^d^	145.34 ± 3.76^a^	138.76 ± 1.39^b^	112.59 ± 2.57^c^
Protein	243.12 ± 2.44^a^	219.57 ± 1.06^b^	196.89 ± 2.37^d^	214.34 ± 0.37^c^
Polysaccharide	106.38 ± 3.53^a^	86.76 ± 0.69^b^	112.09 ± 3.43^a^	74.45 ± 4.52^c^
Ash content	102.20 ± 1.71^b^	112.64 ± 3.26^a^	110.27 ± 1.19^a^	98.31 ± 1.34^c^
Total sugar	309.89 ± 4.36^c^	387.23 ± 3.74^a^	362.77 ± 3.20^b^	248.35 ± 11.07^d^
Reducing sugar	20.13 ± 0.15^a^	12.27 ± 0.14^c^	20.45 ± 0.84^a^	16.43 ± 0.53^b^

Notes: Different letters in the same line indicated that the target parameters of dried L. *decastes* are significantly different (*P* < 0.05). Except for moisture content, which is calculated on a wet basis, all other component contents are calculated on a dry matter basis.

### 3.2 Aroma characteristics of dried L. decastes by *E*-nose

**Fig. 1:**
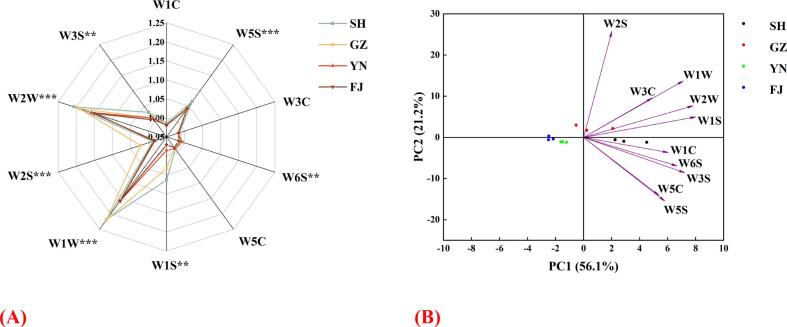
Electronic nose data of dried *L.decastes*. (A) Radar diagram; (B) PCA. The electronic nose sensor array consists of 10 sensors with specific sensitivity: W1C (aromatic compounds), W5S (nitrogen oxides), W3C (amines and aromatic compounds), W6S (hydrogen-containing compounds), W5C (alkanes and aromatic compounds), W1S (short-chain alkanes), W1W (inorganic sulfur and terpenoid compounds), W2S (alcohols, aldehydes, ethers, and ketones), W2W (organic sulfur and terpenoid compounds), and W3S (long-chain alkanes).

The response of the E-nose to dried L. *decastes* was shown in Fig. 1A. The shape of the radar chart indicated that the aroma profiles of the four dried L. *decastes* samples were similar, but the intensity and proportions of various volatile gases varied significantly. The sensors demonstrating the highest response values, in descending order, were W1W, W2W, W1S, and W5S. The results indicated that dried L. *decastes* contained substantial levels of sulfur-containing compounds, terpenoid, short-chain alkanes and nitrogen oxides. These volatiles are characteristic compounds of dried mushroom and have been extensively observed in other dried mushrooms such as *Lentinus edodes* **(**Liu et al., 2022**)***.* Meanwhile, significant variations in signal intensity were observed among the four samples (Fig. 1A). Specifically, sample SH and GZ exhibited markedly higher concentrations of sulfur-containing and terpenoid compounds as compared to the FJ and YN.

Further, the principal component analysis (PCA) was performed to assess the spatial distribution and distance of the dried L. *decastes* aromas. As shown in Fig. 1B, the contribution rate of PC1 was 56.1%, the contribution rate of PC2 was 21.2%, and the total contribution rate was 77.3%. The cumulative contribution rate (77.3%) of PCA indicated that these two principal components represented the main characteristics of the volatile components in dried L. *decastes*, and the sensor data of *E*-nose was high enough to distinguish different mushroom samples **(**Yao et al., 2022**)**. The SH and GZ samples were primarily clustered along the positive PC1 axis and were correlated with all sensors, indicating similar aroma characteristics between the two samples as detected by E-nose (Fig. 1B). Nevertheless, the YN and FJ samples were clustered along the negative PC1 axis (Fig. 1B). Hence, the E-nose coupled with PCA could serve as a useful tool for identification different samples of dried L. *decastes*, which has also been successfully applied to distinguish different dried edible fungi in previous works **(**Gómez et al., 2022**;** Zhou et al., 2015**)**.

### 3.3 Volatile organic compounds (VOCs) identified by GC-IMS and GC–MS

#### 3.3.1 HS-GC-IMS analysis of VOCs

**Fig. 2:**
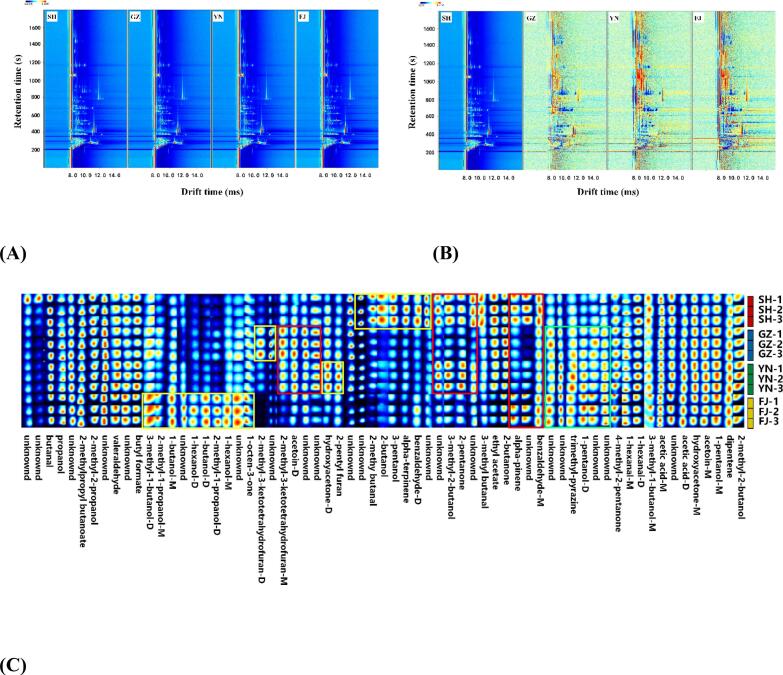
HS-GC-IMS analysis of volatile compounds contained of dried *L.decastes*. (A) Two-dimensional topographic plots; (B) The difference comparison topographic plots; (C) Volatile fingerprints of dried *L.decastes*. M: Monomer form of the compound; D: Dimeric form of the compound.

For HS-GC-IMS analysis, the detection of each component was achieved by combining the retention time (vertical axis) from gas chromatography (GC) with the drift time (horizontal axis) from IMS **(**Yao et al., 2022**)**. The points to the right of the red vertical line (i.e., the normalized ion peak) represent the detected volatile compounds (Fig. 2A). To facilitate a more intuitive comparison of the differences between the four samples, the spectra were deducted that primarily composed of white, blue, and red colors with sample SH used as reference (Fig. 2B). These colors could clearly demonstrate whether the concentration of VOCs in the GZ, FJ, and YN products is equal to (while), lower than (blue), or higher than (red) the corresponding compound contained in the SH **(**Feng et al., 2021**)**. The information revealed a significant number of red and blue points were present in the GZ, YN, and FJ samples, indicating considerable differences in volatile components exhibited among the four mushrooms (Fig. 2B).

The visual fingerprint map was constructed based on the signal peak intensity of each compound, which could further highlight the differences in VOCs among tested samples **(**Yao et al., 2022**)**. As shown in Fig. 2C, each row represents all the signal peaks of mushroom samples, each column represented the signal peaks of the same compound that exhibited in different sample, and the brightness color indicated a high concentration of a compound. A total of GC-IMS 65 signal peaks were detected for all the samples, of which 33 volatile compounds were identified (Fig. 2C). The volatile components of the four dried L. *decastes* samples exhibited significant differences. Specifically, 13 compounds were identified in all four samples, including pentanol, dipentene, acetic acid, 3-hydroxy-2-butanone, hydroxyacetone, *n*-butyraldehyde, *n*-hexanal, *n*-pentanal, *n*-propanol, tert-butanol, 4-methyl-2-pentanone, butyl formate, and isobutyl butyrate (Fig. 2C). Some volatile compounds including 3-hydroxy-2-butanone, *n*-pentanal, and *n*-butyraldehyde were of relatively high contents and had been also identified in dried L. *decastes* samples of other production regions **(****Wu, Wang, et al., 2025****;** Yang et al., 2022**)**. Meanwhile, some compounds were only found with high concentrations in samples of specific production region as indicated by yellow, red, and green rectangles in Fig. 2C. Interestingly, some volatile substances (tert-butanol, 4-methyl-2-pentanone, butyl formate, and isobutyl butyrate) were observed to be abundantly presented in dried L. *decastes* for the first time by GC-IMS, which suggested the great potential of this method on identifying volatiles of mushrooms.

As shown in Fig. 2C, trimethylpyrazine (roasted aroma) and*1*-pentanol (furan oil, fermented aroma) were dominant aroma compounds in YN, FJ, and GZ samples that significantly higher than those in the SH sample. In contrast, benzaldehyde (almond, caramel), α-pinene (lemon, citrus), 3-pentanol (sweet, herbal, nutty), 2-butanol (fruity), and 2-methylbutyraldehyde (moldy, baked) were detected at higher levels only in the SH sample (Fig. 2C). Aldehydes (such as benzaldehyde) are key aroma compounds of dried L. *decastes* mushrooms due to their low odor threshold and high aroma intensity **(**Hou et al., 2021**)**. The YN sample was observed with high levels of 2-pentylfuran (fruity) and hydroxyacetone (caramel), these ketones might impart more fruity aroma to the dried mushroom **(**Zhang et al., 2021**)**. The FJ sample was rich in substances such as 1-octen-3-one (earthy, musty), 1-hexanol (sweet), 2-methyl-1-propanol (fusel oil, alcoholic odor), 1-butanol (alcoholic odor), and 3-methyl-1-butanol (alcoholic odor), with alcohols accounting for a higher proportion. Among these alcohols, 3-methyl-1-butanol was also detected in dried L. *decastes* of other production regions **(**Yang et al., 2016**;** Yang et al., 2022**)**.

#### 3.3.2 HS-SPME-GC-O-MS analysis of volatile compounds

**Fig. 3:**
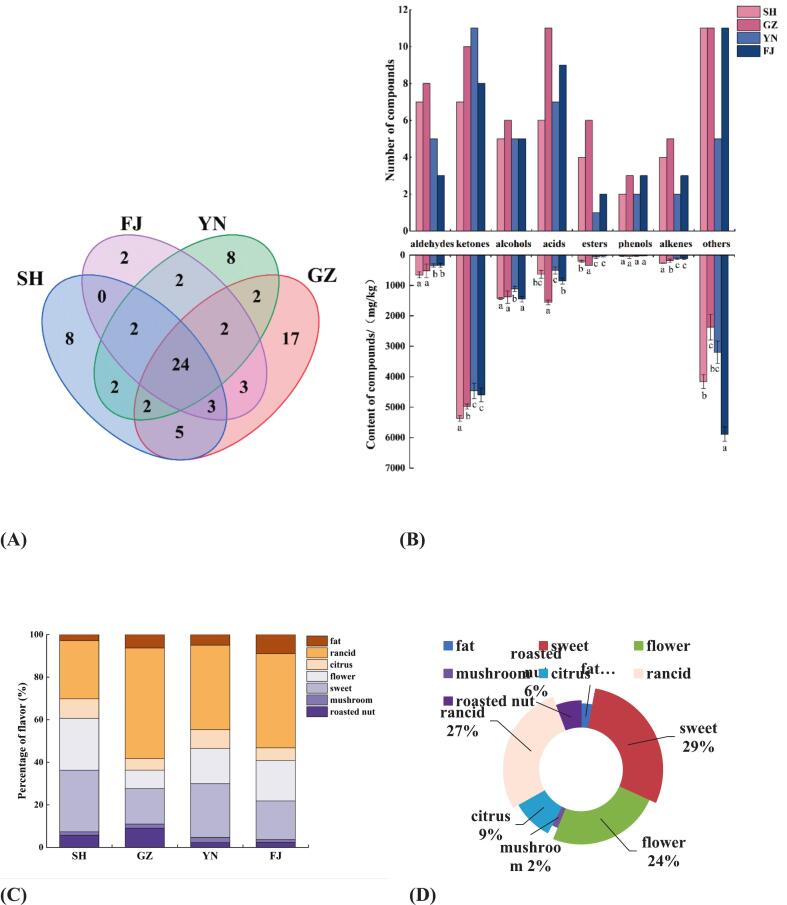
HS-GC-O-MS analysis of volatile compounds contained of dried *L.decastes*. (A) The Venn diagram of the total types of volatile compounds detected by GC–MS; (B) Total types and total contents of volatile compounds detected by GC–MS; (C) The Stack diagram of percentage of the aroma characteristics obtained through GC-O sniffing of dried *L.decastes*; (D) The Outline of fragrance of SH.

A total of 82 VOCs were identified in dried L. *decastes* using HS-SPME-GC-O-MS (**Table S1**), which including aldehydes (9), ketones (14), alcohols (8), acids (13), esters (8), phenols (4), olefins (7), and other compounds (19). It has been reported that aldehydes, ketones, alcohols, and esters significantly contribute to the formation of food flavor characteristics **(**Pei et al., 2016**;** Zhang et al., 2021**)**. In contrast to the GC-IMS results, 46, 60, 44, and 41 compounds were detected in the four samples by HS-GC-O-MS respectively, with 24 common compounds observed in all the four samples (Fig. 3A). In addition, Fig. 3B further visually compared the types and contents of volatile substances in different samples based on the bar chart. The result indicated that the GZ product contains the highest number of distinct compounds. Interestingly, ketones content in all samples significantly exceeded that of other compound categories (Fig. 3B), while these compounds usually had relative low content in other dried mushroom such as *Flammulina velutipes* when compared to fresh specimens **(**Yang et al., 2016**)**. Furthermore, the identified compounds were systematically classified using GC-O combined with aroma profile analysisto further explore the aroma characteristics of dried L. *decastes* (Fig. 3C). The results indicated that the aroma of dried L. *decastes* could generally be categorized into seven notes including fatty (such as nonanal, hexanal, nonanoic acid), rancid (isobutyric acid, isovaleric acid, caproic acid, etc.), citrus (limonene), floral (benzyl alcohol, phenethyl alcohol, etc.), sweet (benzaldehyde, phenylacetaldehyde), mushroom (1-octen-3-ol), and roasted nut (2-*n*-pentylfuran, etc.) aromas. Among them, rancid, sweet and floral notes accounted for the most prominent proportion in its overall aroma composition, constituting the core characteristics of the aroma of dried *L. decastes* (Fig. 3D). The dominant notes were likely attributed to the synergistic effects of aldehydes, ketones, and alcohols, as these compounds were well-known key contributors to mushroom flavor **(**Yang et al., 2022**;** Zhang et al., 2021**)**.

Among detected volatile compounds that mainly contributed to aroma of dried mushroom, aldehydes including benzaldehyde, phenylacetaldehyde, and nonanal were identified in all the four samples (**Table S1**). Benzaldehyde could impart a pleasant almond and caramel aroma to dried L. *decastes* and is the primary aldehyde in these samples, originating from the metabolism of benzoic acid in L. *decastes* **(**Yang et al., 2016**)**. Phenylacetaldehyde is another aldehyde that could impart a honey-like sweet aroma to dried L. *decastes*, and nonanal would contribute a fatty and grassy aroma with a strong flavor that can overlap with other odorants even at low concentrations **(**Zhuang et al., 2016**)**. Ketones were another important aroma compounds detected by GC-O-MS, with 2-nonanone presented the highest proportion that could impart a fresh and green aroma (Fig. 3D and **Table S1**). Additionally, alcohol compounds are important flavor volatiles of mushrooms that usually exhibit similar aroma note (i.e., green, floral, and fatty) to those of aldehydes **(**Pennazza et al., 2013**;** Tietel & Masaphy, 2017**)**. For example, benzyl alcohol is a C_8_ compound and has been identified as a key aroma compound of mushrooms that could impart a sweet and floral aroma **(**Pei et al., 2016**)**. It is noteworthy that the content of 1-octen-3-ol (mushroom alcohol) appeared to be higher than other C_8_ compounds such as 2,3-octanedione, octanol, and octanoic acid detected in dried L. *decastes* (**Table S1**). The mushroom alcohol is an aliphatic unsaturated alcohol and imparts a typical mushroom aroma with extremely low odor threshold (0.01 ppm) **(**Jung et al., 2021**)**. The rancid aroma was significantly represented in all mushroom samples (Fig. 3D and **Table S1**), which had usually been recognized as off-flavors that mainly composed of sulfur-containing compounds and not always been flavor characteristic of dried mushrooms **(**Chun et al., 2020**;** Qin et al., 2020**)**. The high response value of sulfur compound sensors in *E*-nose confirmed the contribution of sulfurous volatiles to the rancid aroma of dried L. *decastes* (Fig. 1). Additionally, short-chain fatty acids (such as butyric acid, valeric acid, hexanoic acid, and octanoic acid) are commonly associated with rancid or soapy odors due to their low odor thresholds **(**Li et al., 2021**)**, which might be another volatile components of the rancid aroma. The types and concentrations of acidic compounds were highly exhibited in dried L. *decastes* samples especially for GZ sample based on the GC-O-MS analysis (**Table S1**).

### 3.4 Taste characteristics of dried L. decastes by E-tongue

**Fig. 4:**
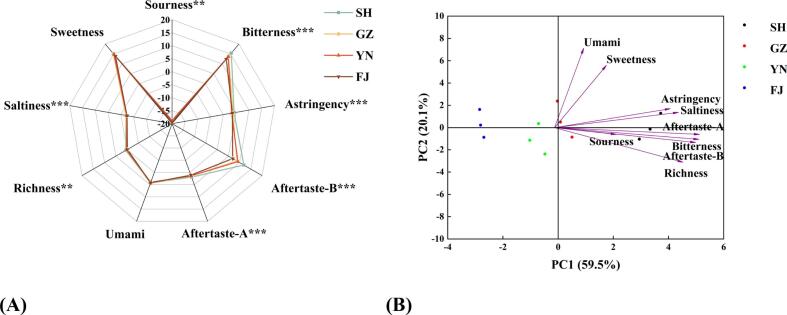
Electronic tongue data of dried *L.decastes*. (A) Radar diagram; (B) PCA.

The taste characteristics of four dried mushroom samples were analyzed using E-tongue with nine taste attributes detected, including sweetness, sourness, saltiness, umami, richness (umami aftertaste), astringency, astringent aftertaste, bitterness, and bitter aftertaste. As shown in Fig. 4A, saltiness and sourness exhibited negative response values, while richness and astringent aftertaste showed weak signals, with values close to or below 0. The umami and sweetness values of dried L. *decastes* were relatively high, while no significant differences observed among the four samples (Fig. 4A). The intensity of the umami flavor in dried L. *decastes* is likely linked to the levels of free amino acids, nucleotides, and related compounds **(**Wang et al., 2020**;** Zheng et al., 2026**)**. In contrast, bitterness and bitter aftertaste exhibited substantial differences (*p* < 0.05) among the four samples with clearly visible variation (Fig. 4A). Meanwhile, the SH product showed significantly higher responses in both bitterness and bitter aftertaste compared to the FJ product. It has been reported that the obvious bitterness in dried L. *decastes* is likely due to the presence of significantly higher levels of bitter amino acids and other bitter compounds **(**Zhang et al., 2026**)**. Additionally, other taste attributes presented relatively low response, indicating they were not representative in the taste profile of dried L. *decastes* (Fig. 4A). Furthermore, PCA was conducted using the sensor response values of taste attributes for the four mushroom samples (Fig. 4B). The first principal component accounts for 59.5% of the variance, and the second accounted for 20.1%, with a cumulative contribution of 79.6%. Overall, there was no overlap between the products. The sample SH clustered along the positive PC1 axis and correlated with all sensors, whereas the YN and FJ aggregated along the negative PC1 axis (Fig. 4B). The result indicated that the four different mushroom samples could be well distinguished by PCA based on the taste characteristics of E-tongue.

### 3.5 Non-volatile substances detected in mushroom samples

Non-volatile compounds, such as free amino acids, organic acids, and nucleotides, are core contributors to the flavor characteristics of mushrooms, with their composition and concentration could directly influence umami, sweetness, and sourness profile **(**Xu et al., 2021**)**. Free amino acids can be divided into four categories in terms of taste characteristics, including umami amino acids (Glu, Asp), sweet amino acids (Thr, Gly, His, Ala, Pro, Ser), bitter amino acids (Met, Arg, Val, Leu, Ile), and tasteless amino acids (Phe, Tyr, Cys, Lys) **(**Du et al., 2024**)**. The total free amino acids content revealed great difference among the four samples (Table 2), which could play crucial role in flavor development and also serve as essential nutritional constituents in mushrooms **(**Zeng et al., 2024**)**. As shown in Table 2, bitter amino acids were the most abundant in dried L. *decastes*, followed by umami, sweet, and tasteless amino acids respectively, consistent with prior findings **(**Qiu et al., 2024**)**. Regarding umami characteristics, *L*-glutamic acid (Glu), a key umami compound, was present at significantly higher levels in SH and FJ samples compared to other amino acids. In addition, glycine (Gly) and alanine (Ala) were relatively abundant among sweet-tasting amino acids, suggesting their potential contribution to the sweetness of dried L. *decastes*. The umami (2288–3864 mg/100 g) and sweetness (3234–4577 mg/100 g) amino acid contents detected in dried L. *decastes* were greatly higher than those observed in dried L. *edodes* (192–506 mg/100 g and 950–1314 mg/100 g respectively) **(**Hou et al., 2021**)**.

**Table 2:** Comparison of amino acid, organic acid and 5′-nucleotides contents of dried L. *decastes*.

Content (mg/100 g)		SH	GZ	YN	FJ
Free amino acid					
Umami	Asp	1361.24 ± 1.96^a^	1145.32 ± 4.45^a^	915.71 ± 6.87^b^	1201.07 ± 1.85^a^
	Glu	2502.95 ± 19.87^a^	1745.00 ± 12.73^c^	1372.99 ± 3.34^d^	1992.16 ± 21.52^b^
	Total	3864.19 ± 21.83^a^	2890.32 ± 17.18^c^	2288.70 ± 10.22^d^	3193.23 ± 23.37^b^
Sweet	Thr	824.00 ± 11.71^a^	704.40 ± 3.78^a^	582.21 ± 4.10^b^	708.90 ± 2.27^a^
	Ser	844.36 ± 12.03^a^	725.57 ± 8.01^b^	595.72 ± 5.38^c^	772.13 ± 5.73^ab^
	Gly	973.1 ± 1.67^a^	834.96 ± 14.77^b^	685.29 ± 10.25^c^	888.08 ± 4.85^ab^
	Ala	951.72 ± 7.05^a^	752.00 ± 3.35^b^	589.87 ± 5.59^c^	817.87 ± 12.43^ab^
	Pro	588.98 ± 5.26^a^	559.97 ± 7.40^ab^	488.71 ± 7.92^bc^	510.29 ± 2.79^ab^
	His	395.46 ± 11.30^a^	353.70 ± 5.05^ab^	292.65 ± 6.83^bc^	366.93 ± 4.47^ab^
	Total	4577.62 ± 49.02^a^	3930.60 ± 42.35^b^	3234.45 ± 40.07^c^	4064.20 ± 32.53^b^
Bitter	Val	774.11 ± 10.57^a^	603.22 ± 8.85^b^	489.80 ± 8.80^c^	615.69 ± 4.57^b^
	Met	1565.84 ± 12.76^b^	1980.41 ± 4.97^a^	1910.34 ± 2.02^a^	1949.24 ± 8.49^a^
	Ile	606.60 ± 8.89^a^	483.67 ± 15.14^b^	393.3 ± 13.92^b^	484.73 ± 5.94^b^
	Leu	980.15 ± 8.75^a^	783.59 ± 1.09^b^	646.23 ± 5.92^c^	788.18 ± 9.32^b^
	Arg	1086.74 ± 5.87^a^	920.92 ± 5.73^a^	695.17 ± 5.53^b^	1116.24 ± 19.47^a^
	Trp	11.72 ± 0.15^b^	9.54 ± 0.14^b^	3.70 ± 0.01^c^	25.44 ± 0.13^a^
	Total	5025.16 ± 47.00^a^	4781.35 ± 35.93^a^	4138.54 ± 37.21^b^	4979.52 ± 47.92^a^
Tasteless	Lys	1112.27 ± 7.47^a^	902.09 ± 4.41^b^	765.74 ± 4.64^c^	994.60 ± 4.95^b^
	Tyr	658.29 ± 7.26^a^	563.49 ± 3.88^bc^	472.44 ± 5.10^c^	553.06 ± 1.69^ab^
	Phe	114.54 ± 6.03^a^	121.09 ± 7.56^b^	103.92 ± 3.16^c^	112.02 ± 3.10^bc^
	Cys	553.91 ± 3.12^a^	466.29 ± 8.36^a^	401.69 ± 10.75^a^	497.95 ± 4.06^a^
	Total	2439.01 ± 23.89^a^	2052.96 ± 24.21^b^	1743.79 ± 23.65^c^	2157.63 ± 13.81^b^
Total free amino acid		15,905.98 ± 141.74^a^	13,655.23 ± 119.67^b^	11,405.48 ± 111.15^c^	14,394.58 ± 117.63^b^
Organic acid					
Oxalic acid		19.04 ± 0.29^b^	15.46 ± 0.10^c^	8.14 ± 0.20^d^	24.65 ± 0.09^a^
Tartaric acid		240.41 ± 2.30^a^	75.28 ± 2.29^c^	91.68 ± 2.60^b^	62.46 ± 3.04^d^
Formic acid		318.17 ± 6.06^a^	121.83 ± 2.97^d^	210.99 ± 3.21^b^	163.69 ± 3.23^c^
Malic acid		2795.34 ± 13.58^c^	2918.15 ± 6.95^b^	3755.49 ± 9.05^a^	2327.79 ± 15.03^d^
Malonic acid		5.68 ± 0.12^c^	5.99 ± 0.09^b^	6.69 ± 0.06^a^	2.87 ± 0.01^d^
Lactic acid		84.94 ± 0.40^b^	52.79 ± 0.18^d^	78.09 ± 0.13^c^	87.86 ± 0.28^a^
Acetic acid		4172.25 ± 16.76^b^	2340.90 ± 17.59^d^	3832.8 ± 8.43^c^	4868.16 ± 23.10^a^
Maleic acid		0.88 ± 0.33^a^	0.37 ± 0.07^b^	0.76 ± 0.08^a^	0.41 ± 0.07^b^
Citric acid		17.51 ± 0.47^a^	10.12 ± 1.30^b^	9.84 ± 0.16^b^	7.81 ± 0.20^c^
Total organic acid		7654.22 ± 40.01^b^	5540.89 ± 31.54^d^	7994.48 ± 23.93^a^	7545.70 ± 45.05^c^
5′-Nucleotides					
5′-CMP		36.85 ± 0.36^a^	18.24 ± 0.37^c^	12.47 ± 0.10^d^	25.78 ± 0.11^b^
5′-AMP		14.86 ± 0.40^a^	7.19 ± 0.42^c^	5.91 ± 0.04^d^	8.24 ± 0.10^b^
5′-UMP		15.00 ± 0.20^a^	9.93 ± 0.19^d^	11.50 ± 0.14^c^	13.50 ± 0.17^b^
5′-GMP		19.65 ± 0.18^a^	17.28 ± 0.21^b^	13.19 ± 0.04^c^	16.95 ± 0.31^b^
Total 5′-nucleotides		86.36 ± 1.14^a^	52.64 ± 1.19^c^	43.07 ± 0.32^d^	64.47 ± 0.70^b^

Notes: Different letters in the same line indicated that the target parameters of dried *L. decastes* are significantly different (*P* < 0.05).

Organic acids are common bioactive compounds in mushrooms and serve as important flavor components, contributing to sour and astringent tastes **(**Du et al., 2024**;** Ribeiro et al., 2008**)**. As shown in Table 2, the YN sample exhibited the highest organic acid content, followed by SH, FJ, and GZ respectively. Specifically, acetic acid was the most abundant (2340–4868 mg/100 g) in dried *L. decastes*, accounting for over 40% of the total organic acids, followed by malic acid (2327–3755 mg/100 g) (Table 2). The relatively high malic acid content in the dried L. *decastes* might contribute to its pronounced umami flavor as malic acid could enhance the umami taste of mushrooms **(**Pei et al., 2014**)**. In addition, 5′-nucleotides are typical flavor compounds that act synergistically with Glu and Asp to enhance umami perception **(**Lin et al., 2020**)**. Herein, four 5′-nucleotides including 5′-CMP, 5′-UMP, 5′-GMP, and 5′-AMP were detected, with 5′-CMP and 5′-GMP revealed to be most abundant in dried *L. decastes* (Table 2). The result was different from major flavor nucleotides of other dried mushroom such as L. *edodes*, which were mainly composed of 5′-CMP and 5′-AMP **(**Zhang et al., 2021**)**. Furthermore, comparison of total contents revealed that sample SH contained the highest content of 5′-nucleotides (86.36 mg/100 g), and YN had the lowest content (43.07 mg/100 g) (Table 2).

### 3.6 Differences in flavor omics of the four mushroom samples by MFA

**Fig. 5:**
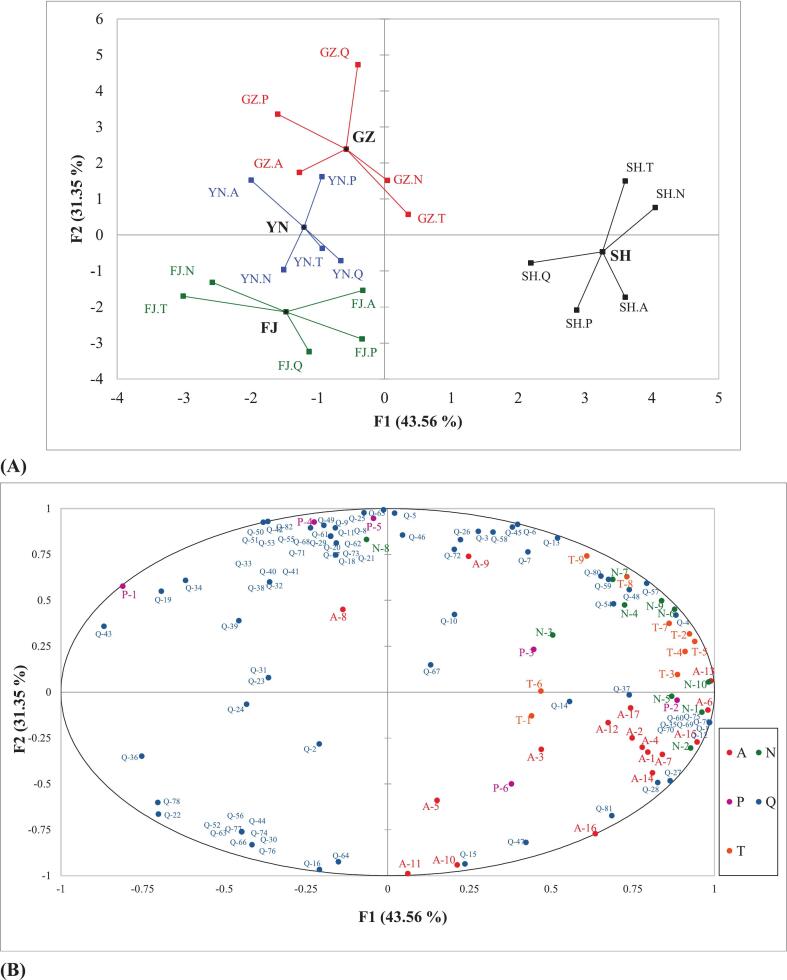
(A) Coordinates of the projected points of the variations of the MFA; (B) Correlations between variables and factors about dried *L.decastes*. The abbreviations Q, P, N, T, A refer to the results identified by GC–MS (the number prefixed by Q is used to replace compounds, corresponding to the number showed in **Table S1**); physicochemical indices (P-1 ∼ P-6: moisture, protein, polysaccharide, ash content, total sugar, reducing sugar); the response values of ten electronic nose sensors (N-1 ∼ N-10: W1C, W5S, W3C, W6S, W5C, W1S, W1W, W2S, W2W, W3S); the response values of nine electronic tongue sensors (T-1 ∼ T-6: sourness, bitterness, astringency, aftertaste-B, aftertaste-A, umami, richness, saltiness, sweetness) and amino acids (A-1 ∼ A-4: umami, sweet, bitter, tasteless); organic acids (A-5 ∼ A-13: oxalic acid, tartaric acid, formic acid, malic acid, malonic acid, lactic acid, acetic acid, maleic acid, citric acid) and 5′-nucleotides (A-14 ∼ A-17: 5′-CMP, 5′-AMP, 5′-UMP, 5′-GMP) data respectively.

MFA examines the relationships among observations, variables, and data tables simultaneously by analyzing them together **(**Sudheer et al., 2018**)**. In this study, MFA was conducted to visualize large datasets through dimensionality reduction based on the results of GC–MS, physical and chemical indices, *E*-nose, E-tongue, and HPLC analysis. The projection of group centers (central points) and the projection of quantitative variables on the MFA plane revealed the relative importance of each group of variables in characterizing the four dried *L. decastes* samples (Fig. 5A). In Fig. 5A, the central points represented the coordinates of the MFA results, while the points connected to them indicated the projection coordinates formed by the variables **(**Li et al., 2021**;** Pagès, 2005**)**. These projections demonstrated that different groups of variables exert varying influences on each central point and the closer these projections are to the central point, the greater the similarity between the descriptions. As shown in Fig. 5A, the sample SH exhibited a distinct spatial aggregation in the first and fourth quadrants, indicating that their non-volatile flavor components (i.e., amino acids, organic acids, and flavor nucleotides) differed markedly from those of the other three samples. In terms of volatile compounds detected by GC–MS (such as aldehydes and alcohols) contributed most significantly to samples differentiation (Fig. 5A). When evaluating the flavor system using multiple quantitative variables, both *E*-tongue (taste characteristics) and E-nose (aroma characteristics) data showed coordinated responses across the four samples (Fig. 5A). Specifically, the projection vectors of the two instruments extended in similar directions within the flavor space, suggesting a correlation between gustatory and olfactory characteristics of dried *L. decastes*. The rotated component variance (RV) coefficient that ranged from 0 to 1was employed to measure the correlation between two sets of variables, which can provide credible information on distinguishing variables and explaining the variance **(**Li et al., 2021**)**. As reported elsewhere, the RV coefficient values of >0.7 are considered as a good level of differentiation **(**Cartier et al., 2006**;** Moelich et al., 2017**)**. The RV coefficient between the E-nose and E-tongue reached 0.945, which demonstrated a high degree of correlation between the two detections (**Table S2**). The result indicated that combination of E-nose and E-tongue could be served as a reliable approach for distinguishing different dried *L. decastes* samples. Additionally, the RV coefficient between physicochemical indicators (including moisture, protein, polysaccharide, ash content, total sugar, and reducing sugar) and non-volatile analysis or GC–MS analysis revealed high value (0.920 and 0.826 respectively) (**Table S2**).

The correlation between variables and factors of the four samples was illustrated in Fig. 5B, with the principal components PC1 and PC2 accounted for 43.56% and 31.35% of the total variance respectively. Combined with Fig. 5A, the GZ and YN samples were located in a similar region of the second quadrant, suggesting the two samples shared comparable flavor characteristics. Further analysis revealed that their flavor profiles were closely associated with several acidic compounds including isobutyric acid (Q-32), isovaleric acid (Q-33), and caproic acid (Q-34) that might contribute to sour and rancid notes of mushrooms (Fig. 5). The proportion of rancid aroma in GZ sample was significantly higher than other samples, this might be associated with several volatile compounds such as hexanal (grassy, tallowy, fatty) and 2-nonanone (heated milk, soapy, green). For the SH sample, it exhibited the highest coordinate value along the F1 axis and showed a strong association with the umami sensor (T-1) of the electronic tongue (Fig. 5). This correlation was likely due to its high concentrations of total 5′-nucleotides (A-14 ∼ A-17:86.36 mg/100 g) and umami amino acids (A-1:3864.19 mg/100 g) as shown in Table 2. Overall, distinct flavor characteristics were exhibited by different dried *L. decastes* samples, with these variations closely linked to contents of volatile and non-volatile flavor compounds.

## 4 Conclusion

The flavor omics of dried L. *decastes* were characterized by integrated utilization of GC-IMS, GC-O-MS, E-nose, E-tongue, and HPLC methods. A total of 82 and 33 VOCs were identified by GC–MS and GC-IMS respectively, with ketones, acids, and aldehydes revealed to be predominant. The volatile aroma compounds detected by olfactometry exhibited floral, sweet, fatty, and rancid notes, with their proportions varying among different samples. The SH sample exhibited significantly different flavor characteristics compared to the others, likely due to its higher concentrations of non-volatile compounds such as amino acids, 5′-nucleotides, and organic acids, which contributed to its enhanced umami and sweet taste. Notably, similar trends were observed between E-tongue and E-nose data, suggesting the combination of these two detections could serve as an effective approach for discriminating different dried L. *decastes* sample. Additionally, this study confirmed that the combination of multiple flavor analysis techniques with multivariate statistical methods such as MFA effectively characterized the flavor profiles of dried L. *decastes*.

## CRediT authorship contribution statement

**Min Sun:** Writing – review & editing, Conceptualization. **Jingbo Shen:** Writing – original draft, Visualization, Data curation. **Yan Liu:** Methodology, Formal analysis. **Tao Feng:** Supervision, Funding acquisition. **Shiqing Song:** Project administration. **Chuang Yu:** Software. **Huatian Wang:** Validation. **Lingyun Yao:** Validation, Resources, Methodology. **Min Deng:** Supervision.

## Declaration of competing interest

The authors declare that they have no known competing financial interests or personal relationships that could have appeared to influence the work reported in this paper.

## Data Availability

Data will be made available on request.
