# Supplementary material for: Flavor omics approach of dried Lyophyllum decastes mushroom using GC-IMS, GC-O-MS, E-nose, E-tongue, and multiple factor analysis
Source: Food Chem X. 2026 Apr 26;36:103925. doi: 10.1016/j.fochx.2026.103925 (PMC13218218; doi:10.1016/j.fochx.2026.103925)
Supplement: Supplementary file 1 — Supplementary material [file mmc1.docx]

**Table S1**

Volatile compounds in SH, GZ, YN and FJ dried *L. decastes* detected by HS-SPME/GC-O-MS

| **NO.** | **Compound Name** | **Odor**^a^ | **RI**^b^ | | **Concentration (mg/kg, mean ± SD, n=4)**^c^ | | | | **Methods**^d^ |
| --- | --- | --- | --- | --- | --- | --- | --- | --- | --- |
|  |  |  | **Calculated** | **Reference** | **SH** | **GZ** | **YN** | **FJ** |  |
|  | **Aldehydeas（9）** |  |  |  | (7) | (8) | (5) | (3) |  |
| Q-1 | Heptaldehyde | fat, citrus, rancid | 1245 | 1217 | 5.7±1.9 | ND | ND | ND | MS,RI |
| Q-2 | 1-Nonanal | fat, citrus, green | 1395 | 1396 | 38.0±2.6 | 45.5±10.8 | 28.0±0.8 | 53.4±4.3 | MS,RI,O |
| Q-3 | 3-Ethylbenzaldehyde | ND | 1444 | ND | 19.5±2.9 | 25.6±1.5 | 22.8±1.8 | ND | MS |
| Q-4 | Benzaldehyde | almond, burnt sugar | 1537 | 1536 | 489.2±27.9 | 409.9±74.1 | 278.6±10.0 | 258.1±30.4 | MS,RI,O |
| Q-5 | Phenylacetaldehyde | hawthorne, honey, sweet | 1654 | 1663 | 57.3±3.0 | 82.5±20.0 | 57.5±1.6 | 45.8±5.3 | MS,RI,O |
| Q-6 | Pyrrole-2-carboxaldehyde | ND | 2035 | 2035 | 7.0±0.6 | 9.5±2.5 | 5.6±0.2 | ND | MS,RI |
| Q-7 | Cocal | ND | 2079 | 2052 | 14.2±1.3 | 23.5±3.5 | ND | ND | MS,RI |
| Q-8 | Hexanal | grass, tallow, fat | 1105 | 1089 | ND | 83.2±5.6 | ND | ND | MS,RI,O |
| Q-9 | Salicylaldehyde | ND | 1732 | 1703 | ND | 9.53±2.5 | ND | ND | MS,RI |
|  |  |  |  |  |  |  |  |  |  |
|  | **Ketones（14）** |  |  |  | (7) | (10) | (11) | (8) |  |
| Q-10 | 5-Methyl-3-hexen-2-one | ND | 1250 | ND | 36.8±6.1 | 47.0±13.7 | 22.2±0.5 | 35.4±9.1 | MS |
| Q-11 | 2-Nonanone | hot milk, soap, green | 1388 | 1388 | 2847.2±164.3 | 3330.8±319.4 | 2870.6±43.6 | 2814.6±95.9 | MS,RI |
| Q-12 | L-menthone | mint | 1473 | ND | 36.1±1.1 | ND | ND | ND | MS |
| Q-13 | 3-Nonen-2-one | ND | 1513 | 1522 | 166.0±8.6 | 181.1±46.8 | 137.2±3.2 | 113.4±8.4 | MS,RI |
| Q-14 | Geranylacetone | magnolia, green | 1850 | 1868 | 40.5±1.4 | 38.6±1.5 | 29.1±0.8 | 37.9±1.9 | MS,RI,O |
| Q-15 | Trans-4-phenyl-3-buten-2-one | ND | 2127 | ND | 924.5±88.3 | 711.9±91.1 | 772.4±34.5 | 993.3±46.1 | MS |
| Q-16 | 6-Methyl-3,5-heptadien-2-one | ND | 1596 | 1602 | 269.0±19.9 | ND | 326.6±8.6 | 531.2±11.9 | MS,RI |
| Q-17 | 2,3-Octanedione | ND | 1322 | 1325 | ND | 7.4±1.8 | 3.9±0.2 | ND | MS,RI |
| Q-18 | 6-Methyl-5-hepten-2-one | ND | 1343 | ND | ND | 50.7±1.3 | ND | ND | MS |
| Q-19 | 2-Decanone | ND | 1491 | 1491 | ND | 32.1±11.4 | 13.0±0.4 | 18.0±0.4 | MS,RI |
| Q-20 | N-methyl-2-pyrrolidone | ND | 1684 | 1678 | ND | 246.2±33.6 | 35.4±2.7 | ND | MS,RI |
| Q-21 | 4-(2-furanyl)-3-Buten-2-one | ND | 1911 | 1879 | ND | 22.6±5.1 | ND | ND | MS,RI |
| Q-22 | 4-Hydroxy-3-methylacetophenone | ND | 2010 | ND | ND | ND | 11.7±1.2 | 14.7±1.0 | MS |
| Q-23 | Isophorone | ND | 1606 | 1607 | ND | ND | 14.8±0.4 | ND | MS,RI |
|  |  |  |  |  |  |  |  |  |  |
|  | **Alcohols（8）** |  |  |  | (5) | (6) | (5) | (5) |  |
| Q-24 | Diacetone alcohol | ND | 1368 | 1351 | 1054.0±57.1 | 1191.3±115.0 | 1019.5±35.1 | 1221.2±97.4 | MS,RI |
| Q-25 | 1-Octen-3-ol | mushroom | 1438 | 1452 | 29.3±8.2 | 56.9±2.8 | 32.9±1.2 | 20.6±5.4 | MS,RI,O |
| Q-26 | 2-Ethylhexanol | rose, green | 1478 | 1490 | 9.5±1.5 | 24.3±7.7 | ND | ND | MS,RI,O |
| Q-27 | Benzyl alcohol | sweet, flower | 1879 | 1898 | 256.9±12.7 | 94.7±24.3 | 96.6±4.3 | 156.3±10.5 | MS,RI,O |
| Q-28 | Phenethyl alcohol | honey, spice, rose, lilac | 1914 | 1872 | 153.1±6.1 | 100.3±8.1 | 93.8±3.3 | 122.9±6.1 | MS,RI,O |
| Q-29 | Nerolidol | wood, flower, wax | 2027 | 2050 | ND | 71.4±9.7 | ND | ND | MS,RI |
| Q-30 | 2-(Vinyloxy)ethanol | ND | 2453 | ND | ND | ND | ND | 27.7±17.6 | MS |
| Q-31 | 1-Octanol | chemical, metal, burnt | 1545 | 1550 | ND | ND | 19.1±0.3 | ND | MS,RI |
|  |  |  |  |  |  |  |  |  |  |
|  | **Acids（13）** |  |  |  | (6) | (11) | (7) | (9) |  |
| Q-32 | Isobutyric acid | rancid, butter, cheese | 1563 | 1544 | 38.1±1.5 | 94.2±20.3 | 34.3±1.9 | 39.4±2.5 | MS,RI,O |
| Q-33 | Isovaleric acid | sweat, acid, rancid | 1664 | 1660 | 332.2±12.3 | 875.8±177.9 | 258.2±57.4 | 381.0±80.2 | MS,RI,O |
| Q-34 | Hexanoic acid | sweat | 1838 | 1831 | 103.1±3.5 | 319.6±64.2 | 176.3±6.0 | 208.2±10.7 | MS,RI,O |
| Q-35 | Pterin-6-carboxylic acid | ND | 2095 | ND | 13.9±8.9 | ND | ND | ND | MS |
| Q-36 | Nonanoic acid | green, fat | 2155 | 2211 | 16.1±0.9 | 57.9±4.7 | 38.3±4.7 | 96.3±2.9 | MS,RI,O |
| Q-37 | Benzoic acid | urine | 2435 | 2448 | 103.5±10.1 | 86.3±9.2 | 49.5±1.0 | 79.7±0.8 | MS,RI |
| Q-38 | Butyric Acid | rancid, cheese, sweat | 1623 | 1652 | ND | 51.8±9.8 | ND | 19.3±0.4 | MS,RI,O |
| Q-39 | Valeric acid | sweat | 1732 | 1734 | ND | 53.5±2.5 | ND | 32.8±2.2 | MS,RI,O |
| Q-40 | 2-Methyl butyric acid | cheese, sweat | 1742 | 1711 | ND | 36.4±0.8 | ND | ND | MS,RI,O |
| Q-41 | Tiglic acid | ND | 1843 | 1867 | ND | 77.0±8.9 | ND | ND | MS,RI |
| Q-42 | Heptanoic acid | ND | 1944 | 1950 | ND | 30.2±1.6 | 14.5±0.8 | ND | MS,RI |
| Q-43 | Octanoic acid | sweat, cheese | 2050 | 2039 | ND | 28.9±2.1 | 17.7±0.8 | 22.4±0.6 | MS,RI,O |
| Q-44 | Trans-2, 3-dimethacrylic acid | ND | 1843 | 1835 | ND | ND | ND | 41.3±0.9 | MS,RI |
|  |  |  |  |  |  |  |  |  |  |
|  | **Esters（8）** |  |  |  | (4) | (6) | (1) | (2) |  |
| Q-45 | Tulipalin A | ND | 1711 | ND | 126.3±7.3 | 163.7±44.0 | 115.1±2.8 | ND | MS |
| Q-46 | Methyl salicylate | peppermint | 1788 | 1751 | 12.3±1.2 | 55.6±45.6 | ND | ND | MS,RI |
| Q-47 | γ-Nonanolactone | coconut, peach | 2039 | 2008 | 33.9±4.0 | ND | ND | 36.3±0.8 | MS,RI |
| Q-48 | C12E8 | ND | 2321 | ND | 27.2±17.2 | 25.2±13.7 | ND | ND | MS |
| Q-49 | 4-Hexanolide | coumarin, sweet | 1717 | 1726 | ND | 27.3±6.0 | ND | ND | MS,RI |
| Q-50 | DL-Pantolactone | ND | 2040 | 2034 | ND | 58.8±2.8 | ND | ND | MS,RI |
| Q-51 | Methyl palmitate | ND | 2203 | 2218 | ND | 9.0±0.3 | ND | ND | MS,RI |
| Q-52 | Octyl formate | ND | 1545 | ND | ND | ND | ND | 24.7±0.2 | MS |
|  |  |  |  |  |  |  |  |  |  |
|  | **Phenols（4）** |  |  |  | (2) | (3) | (2) | (3) |  |
| Q-53 | O-cresol | phenol | 2002 | 1992 | 24.1±1.7 | 63.7±2.1 | 28.7±1.1 | 22.0±0.8 | MS,RI,O |
| Q-54 | Phenol | phenol | 2008 | 2037 | 18.3±1.4 | 18.2±2.2 | 12.6±0.0 | 13.9±0.8 | MS,RI,O |
| Q-55 | 1-Naphthol | ND | 1862 | ND | ND | 19.0±3.4 | ND | ND | MS |
| Q-56 | 4-Propoxyphenol | ND | 1807 | ND | ND | ND | ND | 201.7±38.4 | MS |
|  |  |  |  |  |  |  |  |  |  |
|  | **Alkenes（7）** |  |  |  | (4) | (5) | (2) | (3) |  |
| Q-57 | (+)-Dipentene | citrus, mint | 1207 | ND | 172.9±8.9 | 160.4±22.6 | 118.4±5.6 | 99.6±0.5 | MS,O |
| Q-58 | Styrene | balsamic, gasoline | 1267 | 1267 | 5.4±0.7 | 6.3±0.9 | 4.7±0.2 | 4.4±0.4 | MS,RI |
| Q-59 | (-)-α-Cedrene | ND | 1579 | 1587 | 19.0±0.7 | 20.0±2.2 | ND | ND | MS,RI |
| Q-60 | (-)-Isoledene | ND | 1816 | ND | 36.1±0.8 | ND | ND | ND | MS |
| Q-61 | β-Bisabolene | balsamic | 1725 | 1741 | ND | 28.5±15.7 | ND | ND | MS,RI |
| Q-62 | (+)-Delta-cadinene | thyme, medicine, wood | 1760 | 1711 | ND | 8.2±0.0 | ND | ND | MS,RI |
| Q-63 | (+)-Calarene | ND | 1602 | 1610 | ND | ND | ND | 27.0±18.2 | MS,RI |
|  |  |  |  |  |  |  |  |  |  |
|  | **Others（19）** |  |  |  | (11) | (11) | (5) | (11) |  |
| Q-64 | 2-Methoxyfuran | ND | 1148 | ND | 3613.5±345.9 | 2563.6±763.3 | 3012.1±74.4 | 5333.3±187.6 | MS |
| Q-65 | 2-Pentylfuran | green bean, butter | 1233 | 1234 | 25.7±1.1 | 36.7±9.4 | 29.9±1.5 | 18.4±1.9 | MS,RI,O |
| Q-66 | 2,5-Dimethyltetrahydrofuran | ND | 1626 | ND | ND | ND | ND | 9.7±0.2 | MS |
| Q-67 | 2-Picoline | sweat | 1224 | 1219 | 22.4±2.4 | 34.1±7.7 | ND | 27.4±0.8 | MS,RI |
| Q-68 | 3-Picoline | ND | 1304 | 1284 | ND | 28.8±1.7 | ND | ND | MS,RI |
| Q-69 | 4-Picoline |  | 1303 | 1298 | 38.0±6.6 | ND | ND | ND | MS,RI |
| Q-70 | 2-Phenylpyridine | ND | 2258 | ND | 36.7±2.1 | ND | ND | ND | MS |
| Q-71 | 3-Phenylpyridine | ND | 2258 | 2293 | ND | 27.6±0.2 | ND | ND | MS,RI |
| Q-72 | 2-Acetyl pyrrole | nut, walnut, bread | 1977 | 1967 | 83.3±3.0 | 201.8±39.4 | ND | 22.4±1.0 | MS,RI,O |
| Q-73 | 2,5-Dimethyl pyrazine | cocoa, roasted nut, roast beef, medicine | 1328 | 1328 | ND | 30.6±11.8 | ND | ND | MS,RI,O |
| Q-74 | 2,6-Dimethyl pyrazine | roasted nut, cocoa, roast beef | 1334 | 1335 | ND | ND | ND | 15.2±2.4 | MS,RI |
| Q-75 | 3-Ethyl-2,5-dimethylpyrazine | potato, roast | 1445 | 1471 | 19.3±5.9 | ND | ND | ND | MS,RI |
| Q-76 | o-Toluidine | ND | 1813 | 1800 | 16.8±0.7 | ND | 20.0±0.2 | 146.8±94.1 | MS,RI |
| Q-77 | 1,4-Diaminobutane | ND | 1466 | 1434 | ND | ND | ND | 9.6±0.4 | MS,RI |
| Q-78 | Naphthalene | tar | 1757 | 1765 | ND | ND | 16.7±0.8 | 17.2±0.9 | MS,RI |
| Q-79 | Indole | mothball, burnt | 2460 | 2465 | 64.5±4.1 | ND | ND | ND | MS,RI |
| Q-80 | DL-Menthol | ND | 1635 | 1652 | 100.1±2.2 | 103.4±28.9 | 67.0±1.9 | 67.1±6.6 | MS,RI |
| Q-81 | Benzeneacetonitrile | ND | 1940 | 1947 | 35.9±1.4 | ND | ND | 24.0±1.9 | MS,RI |
| Q-82 | 1-Methylnaphthalene | ND | 1868 | 1884 | ND | 17.4±0.6 | ND | ND | MS,RI |

^a^, Odor: Determined by reference and odor quality at the sniffing mouth.

^b^, RI means retention index calculated using n-alkanes C_7_–C_30_ as internal standard on HP-Innowax column and used for compound identification through compare with the reference values (*https://webbook.nist.gov/chemistry/*).

^c^, SH，GZ，YN，FJ represents different product names. Each value was expressed as mean ± SD (n=4) and The concentrations of volatile compounds were determined by comparing with the concentrations of internal standard (1,2-dichlorobenzene). ND means not detected.

^d^, Methods: MS was identified by matching with NIST 20.0 mass spectrometry library. RI indicates that the RI value is identified by comparing the calculated RI value with the RI value in the reference. O refers to the identification by smelling mouth perception.

**Table S2**

RV coefficients for the correlation of variables obtained with Q, P, N, T and A of dried *L. decastes*.

|  | Q | P | N | T | A |
| --- | --- | --- | --- | --- | --- |
| Q | 1.000 | 0.826 | 0.726 | 0.620 | 0.678 |
| P |  | 1.000 | 0.602 | 0.557 | 0.920 |
| N |  |  | 1.000 | 0.945 | 0.688 |
| T |  |  |  | 1.000 | 0.622 |
| A |  |  |  |  | 1.000 |

Q: Identified VOCs using GC-MS shown in **Table S1**.

P: Moisture, protein, polysaccharide, ash content, total sugar, reducing sugar data shown in **Table 1**.

N: The response values of ten electronic nose sensors are shown in **Fig. 1**.

T: The response values of nine electronic tongue sensors are shown in **Fig. 4**.

A: Amino acids, organic acids and 5′-nucleotides data are shown in **Table 2**.
